# Supplementary figures and images for: Proton pump inhibitor use increases the risk of peritonitis in peritoneal dialysis patients
Source: PLoS One. 2019 Nov 7;14(11):e0224859. doi: 10.1371/journal.pone.0224859 (PMC6837385; doi:10.1371/journal.pone.0224859)

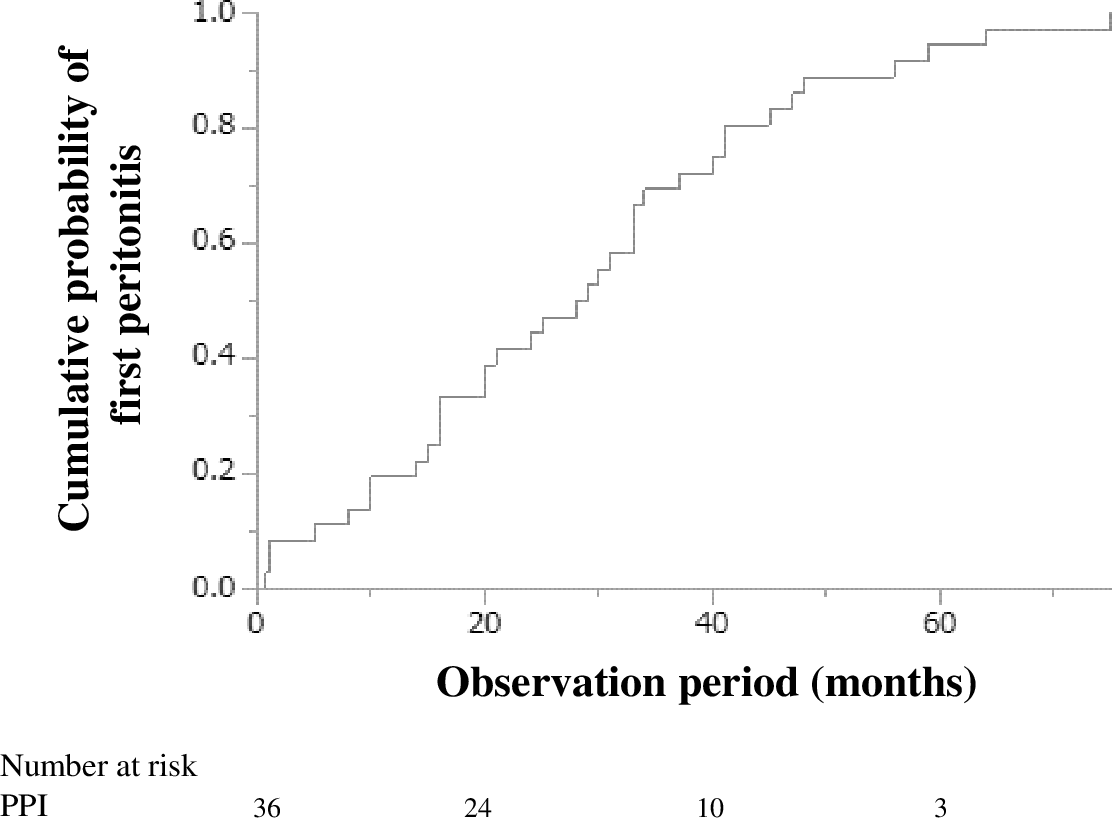

Supplement: S1 Fig — (TIF) [file pone.0224859.s001.tif]
