# Supplementary material for: Proton pump inhibitor use increases the risk of peritonitis in peritoneal dialysis patients
Source: PLoS One. 2019 Nov 7;14(11):e0224859. doi: 10.1371/journal.pone.0224859 (PMC6837385; doi:10.1371/journal.pone.0224859)
Supplement: Attachment — Submitted filename: 20191002_plosone_revise_comments.docx [file pone.0224859.s003.docx]

フォームの始まり

5. Review Comments to the Author
When submitting your revision, we need you to address these additional requirements.

Please ensure that your manuscript meets PLOS ONE's style requirements, including those for file naming. The PLOS ONE style templates can be found at

http://www.journals.plos.org/plosone/s/file?id=wjVg/PLOSOne_formatting_sample_main_body.pdf and http://www.journals.plos.org/plosone/s/file?id=ba62/PLOSOne_formatting_sample_title_authors_affiliations.pdf

1. In the ethics statement in the manuscript and in the online submission form, please provide additional information about the patient records used in your retrospective study. Specifically, please ensure that you have discussed whether all data/tissue samples were fully anonymized before you accessed them and/or whether the IRB or ethics committee waived the requirement for informed consent. If patients provided informed written consent to have data from their medical records used in research, please include this information.

We confirm that all data were fully anonymized and that the ethics committee waived the requirement for obtaining informed consent from the patient.

We changed the following sentence in the second paragraph of the “Study population and data source” subsection within the “Materials and methods” section:

From “Obtaining informed consent from the patients was not required because of the retrospective nature of this study. The study protocol and consent procedure were approved by the respective ethics committees (approval number: 29-12-01).”

To “All data were fully anonymized and that the ethics committee (approval number: 29-12-01) waived the requirement for obtaining informed consent from the patient because of the retrospective nature of this study.”

We also ask that you please provide the full name of the institutional review board(s) or the ethics committees that reviewed and approved this study.

The ethics committee included the following members: Atsushi Kamiya, Minoru, Kadoi, and Hiroshi Miyazawa from Narita Memorial Hospital.

2. We noticed minor instances of text overlap with the following previous publication(s), which need to be addressed:

https://bmcinfectdis.biomedcentral.com/articles/10.1186/s12879-019-4300-0

The text that needs to be addressed involves the abstract.

In your revision please ensure you cite all your sources (including your own works), and quote or rephrase any duplicated text outside the methods section. Further consideration is dependent on these concerns being addressed.

We have modified our abstract as follows, in order to avoid overlap with the previous publication:

Peritonitis is a major and the most significant complication of peritoneal dialysis (PD). Although some predictors of peritonitis in PD patients are known, the association between proton pump inhibitor (PPI) use and peritonitis has not been characterized. Here, we examined whether PPI use is a risk factor for the development of peritonitis, based on a single-center retrospective analysis of 230 consecutive Japanese PD patients at Narita Memorial Hospital. We assessed the association between PPI use and subsequent first episode of peritonitis using multivariate Cox proportional hazards models, following adjustment for clinically relevant factors. The median follow-up period was 36 months (interquartile range, 19-57 months). In total, 86 patients (37.4%) developed peritonitis. Analysis with multivariate Cox proportional hazards models revealed the following significant predictors of peritonitis: PPI use (adjusted hazard ratio [HR]  = 1.72, 95% confidence interval [CI]: 1.11-2.66; P = 0.016) and low serum albumin level (per g/dl adjusted HR  =0.59, 95% CI: 0.39-0.90; P = 0.014). Thus, PPI use was independently associated with PD-related peritonitis. The results suggest that nephrology physicians should exercise caution when prescribing PPIs for PD patients.

3. Please include captions for your Supporting Information files at the end of your manuscript, and update any in-text citations to match accordingly. Please see our Supporting Information guidelines for more information: <http://journals.plos.org/plosone/s/supporting-information>.

We have included the captions for our Supporting Information files at the end of our manuscript, after the references.

Please use the space provided to explain your answers to the questions above. You may also include additional comments for the author, including concerns about dual publication, research ethics, or publication ethics. (Please upload your review as an attachment if it exceeds 20,000 characters)
Thank you for your comments. We have revised our paper accordingly.

Reviewer #1: Maeda S et al explored the association between proton pump inhibitor use and peritonitis using single-center retrospective cohort in their hospital. As a total of 230 peritoneal dialysis patients in the past 20 years have been enrolled, this study is well designed as retrospective cohort study. However I think this work is suitable for being published in PLOS ONE, it cannot be accepted in its original form. I have a several queries such as unclear causal association between PPI use and the occurrence of peritonitis.

We thank the reviewer for these valuable suggestions. We have revised our paper accordingly.

1) The authors define PPI group as ` The patients who took PPI at least 1 week. '. I think it does not include the information about the timing when PPI was taken.

Patients who took any PPI for at least 1 week continuously were included in the PPI group, whereas the remaining patients were categorized into the non-PPI group, as previously reported [14]. In those who developed peritonitis in the PPI group, only the patients who took PPI before developing peritonitis were included in the PPI group.

We modified the sentence of the 2^nd^ paragraph in the “Exposure and outcomes” section as follows:

“Patients who took any PPI for at least 1 week continuously were included in the PPI group, whereas the remaining patients were categorized into the non-PPI group, as previously reported [14]. In those who developed peritonitis in the PPI group, only the patients who took PPI before developing peritonitis were included in the PPI group. We also obtained information regarding the use of H2RA, which was defined as at least 1 week continuously of prescribed H2RA. PPIs included the following drugs: omeprazole, esomeprazole, lansoprazole, rabeprazole, or vonoprazan. H2RA included the following drugs: cimetidine, ranitidine, or famotidine.”

2) Furthermore, this definition does not clarify whether peritonitis was developed within the period the patients took PPI.

Among the total of 41 patients who developed peritonitis in the PPI group, 36 (87.8%) patients developed peritonitis during the period of PPI use. The remaining 5 (12.2%) patients developed peritonitis after discontinuation of PPI, but these patients took H2RA instead of PPI. This suggests that the continued anti-acid effect might predispose the patients to development of peritonitis.

We added the following sentence in the first paragraph of the “Peritonitis incidence” subsection within the “Outcome data” section:

“Among the total of 41 patients who developed peritonitis in the PPI group, 36 (87.8%) developed peritonitis during the period of PPI use. The remaining 5 (12.2%) patients developed peritonitis after discontinuation of PPI, but these patients had taken H2RA instead of PPI.“

3) The authors assume intestinal flora change and bacterial translocation due to oral PPI as the mechanism of peritonitis development. However, logical consistency is insufficient if peritonitis had not occurred within the period when PPI had been prescribed.
The authors should clarify whether the patients were suffered from peritonitis during the period of taking PPI.
Among the total of 41 patients who developed peritonitis in the PPI group, only 5 (12.2%) patients developed peritonitis after discontinuation of PPI. However, these patients took H2RA after discontinuation of PPI; therefore, peritonitis might have developed because of the effect of gastric acid suppression by H2RA, which led to continued changes in the intestinal flora and bacterial translocation.

We added the following sentence in the first paragraph of the “Peritonitis incidence” subsection within the “Outcome data” section:

“Among the total of 41 patients who developed peritonitis in the PPI group, 36 (87.8%) developed peritonitis during the period of PPI use. The remaining 5 (12.2%) patients developed peritonitis after discontinuation of PPI, but these patients had taken H2RA instead of PPI.“

Reviewer #2: Dr. Maeda S et al. reported the association between proton pump inhibitor (PPI) use and the risk of peritonitis in peritoneal dialysis patients in the retrospective single center cohort. In general, the work is interesting, although there are several deficiencies that need to be addressed before it would be suitable for publication, including:

We thank the reviewer for these valuable suggestions. We have revised our paper accordingly.

1. The authors had defined patients who took any PPI for at least 1 week as the PPI group. How many patients had taken any PPI at onset of peritonitis? The authors should show the Kaplan-Meier curve of patients who took any PPI at onset of peritonitis.

Among the total of 41 patients who developed peritonitis in the PPI group, 36 (87.8%) developed peritonitis during the period of PPI use. The remaining 5 (12.2%) patients developed peritonitis after discontinuation of PPI, but these patients took H2RA instead of PPI. In accordance with the reviewer’s comments, we included the Kaplan-Meier curve of patients who took any PPI at the onset of peritonitis as a supplemental figure.

We added the following sentence in the first paragraph of the “Peritonitis incidence” subsection within the “Outcome data” section:

“Furthermore, the cumulative probabilities of the first episode of peritonitis in those who took PPI at the onset of peritonitis are shown in S1 Fig.”

2. The authors should describe the usage of previous/ongoing immunosuppression in Table 1.

We obtained information regarding the usage of immunosuppression (previous/ongoing use); this information was included in Table 1. Eighteen patients in the study used any immunosuppression, and the rate of usage did not significantly differ between the two groups (P=0.152). Furthermore, we added “use of immunosuppression” into covariates for univariate and multivariate CPH models. Use of immunosuppression was not identified as a risk factor for peritonitis, as Table 3.

3. The observational, nonrandomized design does not permit to establish a causality link between treatment with PPI and outcomes. The authors should well describe the possibility that PPI use may just be a confounding factor for other, unknown variables.in limitation.

We inserted the following sentence in the limitation.

“Fifth, because of the retrospective nature of the present study, we could not determine the basis for starting PPI treatment, and therefore could not establish a causal link between PPI treatment and peritonitis.”

Furthermore, we changed the following sentence of the last paragraph under the “Discussion” section:

From “Second, we were able to assess the relationship between the intensive exposure of PPI and peritonitis, which allowed us to confirm the causal relationship between PPI and peritonitis.”

To “Second, we were able to assess the relationship between intensive exposure to PPI and peritonitis, which suggested a significant relationship between PPI treatment and peritonitis.”

Reviewer #3: In this manuscript, the authors evaluated the association of PPI use and peritonitis, and they found that PPI use was associated with increased risk of peritonitis. This paper must be the first one showing the risk of PPI for peritonitis as the authors say and might be very important information in clinical practice for the PD patients, however there seems to be several points should be revised.

We thank the reviewer for these valuable suggestions. We have revised our paper accordingly.

1. The authors defined the PPI group as the patients who took any PPI for at least 1 week and did not define the timing of taking the medicine. Please explain why the authors defined the period of prescription as 1 week and if you have some articles you used as reference, please specify them.

In the present study, PPI use was defined as patients who took any PPI for at least 1 week, as previously reported [14]; thus, the steady maximum mean percentage time of gastric pH >4 was observed after taking PPI for 1 week.

2. Patients taking steroid or immunosuppressant are vulnerable to infection. Please describe the number of patients taking steroid or immunosuppressant, if you have data.

We obtained information regarding the usage of immunosuppression (previous/ongoing use); this information was included in Table 1.

Eighteen patients in the study used any immunosuppression, and the rate of usage did not significantly differ between the two groups (P=0.152). Furthermore, we added “use of immunosuppression” into covariates for univariate and multivariate CPH models. Use of immunosuppression was not identified as a risk factor for peritonitis, as Table 3.

3. The reason why the association between H2-receptor antagonist (H2RA) use and peritonitis was not significant might be due to the small sample size. Please clarify the number of the patients taking H2RA in Table 1.

We clarified the number of the patients taking H2RA in Table 1.

Furthermore, we modified the text of the 2^nd^ paragraph in the “Exposure and outcomes” section as follows:

“Patients who took any PPI for at least 1 week continuously were included in the PPI group, whereas the remaining patients were categorized into the non-PPI group, as previously reported [14]. In those who developed peritonitis in the PPI group, only the patients who took PPI before developing peritonitis were included in the PPI group. We also obtained information regarding the use of H2RA, which was defined as at least 1 week of prescribed H2RA. PPIs included the following drugs: omeprazole, esomeprazole, lansoprazole, rabeprazole, or vonoprazan. H2RA included the following drugs: cimetidine, ranitidine, or famotidine.”

Reviewer #4: In the manuscript entitled “Proton pump inhibitor use increases the risk of peritonitis in peritoneal dialysis patients”, authors conducted a retrospective PD patient study to assess the risk of using PPI against peritonitis. The study is well designed and provides good recommendation for the nephrologists. There are some issues that need to be addressed before publishing.

We thank the reviewer for these valuable suggestions. We have revised our paper accordingly.

1) It is not clear where the H2RA patient cohort is coming from? Please make it clear and include the data in Figure 1

In the present study, we focused on the relationship between PPI use and peritonitis. Therefore, we presume that it might be confusing to include information about H2RA in Figure 1. Instead, we have presented this information in Table 1. We request the reviewer to consider whether this modification is sufficient.

2) In the table 2 make it clear that Peritonitis, peritoneal disfunction etc are the reasons for PD retrieval. Because of the way the table is structured, it took some effort to understand. Make some font changes, or tab difference to make it easy to follow

We modified the structure of Table 2 in accordance with the reviewer’s recommendation.

Namely, we added “Peritonitis incidence” under “PD retrieval”, and added details about “PD withdrawal” from “Other outcomes
 to “PD retrieval”.

Furthermore, we modified a few sentences under the “Outcome data” section in the main text to correspond to the changes made in Table 2, which are as follows.

We added “PD retrieval” above “Peritonitis incidence”, and “PD withdrawal” above “Other outcomes”.

According the abovementioned changes in Table, we modified the text, as follows.

PD withdrawal

PD withdrawal occurred in 57 (78.1%) and 122 (77.7%) patients in the PPI and non-PPI groups, respectively. Among the causes of PD withdrawal, peritonitis occurred in 11 (19.3%) and 17 (13.9%) patients, peritoneal dysfunction in 15 (26.3%) and 40 (32.8%), impairment in ADLs in 3 (5.3%) and 12 (9.8%), and renal transplantation in 3 (5.3%) and 2 (1.6%) patients of the PPI and non-PPI groups, respectively (P=0.664).

Other outcomes:

During the observation period, 10 (13.7%) and 19 (12.1%) patients in the PPI and non-PPI groups, respectively, had mortality events of all causes, indicating that the cause of death was not different between the two groups (P=0.434; Table 2).

3) In this study, baseline data from the patients show that serum albumin from PPI and non-PPI groups are comparable. It is known that inflammation can cause hypoalbuminemia. In other studies where they predicted serum albumin as risk factor, the base serum level at admission was lower. In the current study, it is not clear the low serum albumin is really a predictor or the effect of inflammation, please comment and clarify.

Various factors, such as inflammation or malnutrition, might lead to hypoalbuminemia. The differences in albumin levels between patients in the present study and those in previous studies suggest that the conditions or underlying diseases involved in kidney failure might be different among the studied patients. Therefore, our results should be validated in other cohorts.

We modified the following text in the Discussion.

“Hypoalbuminemia in patients with renal failure is multifactorial and may result from malnutrition or inflammation, and it is possible that the increased risk for peritonitis in hypoalbuminemic patients relates to an underlying inflammatory state [8]. In the future, it should be assessed whether hypoalbuminemia is a true predictor or simply the effect of inflammation, by analysis of another cohort involving patients with various clinical characteristics; however, the present study suggests that patients with hypoalbuminemia should be carefully managed for peritonitis.”

4) A minimum treatment of 1 week with PPI was included in the PPI group. Was the treatment continuous, or intermittent?

Patients who took any PPI for at least 1 week continuously were included in the PPI group.

We modified the sentence in the 2^nd^ paragraph of the “Exposure and outcomes” section as follows:

“Patients who took any PPI for at least 1 week continuously were included in the PPI group, whereas the remaining patients were categorized into the non-PPI group, as previously reported [14].”

5) Authors often compared with another meta analysis and commented that the current work has more sample number. It is not true. Please correct the facts contextually.

We apologize for this lack of clarity. We meant to indicate that the abovementioned meta-analysis of 378 patients might be of insufficient size to evaluate the relationship between PPI and PD. Therefore, the relationship should be confirmed in additional studies.
